# Ice versus lidocaine 5% gel for topical anaesthesia of oral mucosa – a randomized cross-over study

**DOI:** 10.1186/s12871-019-0902-8

**Published:** 2019-12-16

**Authors:** Nishma Hindocha, Filip Manhem, Emmanuel Bäckryd, Mats Bågesund

**Affiliations:** 1 Department of Ear, Nose, Throat and Maxillofacial Surgery, Zealand University Hospital, Koege, Denmark; 2 Public Dental Service Östergötland, Linköping, Region Östergötland, and Department of Biomedical and Clinical Sciences, Linköping University, Linköping, Sweden; 3 Pain and Rehabilitation Center, and Department of Medical and Health Sciences, Linköping University, Linköping, Sweden; 4 Centre for Orthodontics and Paediatric Dentistry, Norrköping, Public Dental Service Östergötland, Region Östergötland, and Department of Biomedical and Clinical Sciences, Linköping University, Linköping, Sweden

**Keywords:** Administration, Topical, Anaesthesia, Ice, Lidocaine

## Abstract

**Background:**

Topical anaesthesia is important to optimize pain control during dental injection. Our aim was to describe a new simple method for topical anaesthesia of oral mucosa and to compare the effectiveness of ice and lidocaine 5% gel for topical anaesthesia of oral mucosa.

**Methods:**

A total of 40 patients aged 10.7–19.5 years were included. The side and method of application were both randomized. Heart rate was recorded, and discomfort and pain were evaluated with a visual analogue scale (VAS). A paired t-test was used to compare mean values, a chi^2^ test was used to compare proportions, and a Pearson correlation test was used to examine correlations between variables.

**Results:**

When ice was used, buccal injection VAS pain was rated lower (*p* = 0.044), and VAS discomfort was rated higher (*p* = 0.001), in comparison to when lidocaine 5% gel was used. There was no significant difference in relative heart rate change between ice and lidocaine 5% gel at either needle stick or injection. Lidocaine 5% gel produced a relative heart rate reduction after palatal injection (0.99 ± 0.06) while buccal injection produced an increased relative heart rate (1.02 ± 0.08) (*p* = 0.010). Unpleasant taste was more frequently reported when lidocaine 5% gel was used (*p* = 0.025). An application time of 1 min was sufficient for both ice and lidocaine 5% gel to achieve pain reduction from needle stick in buccal mucosa.

**Conclusion:**

The cheap and readily available described method using ice for topical anaesthesia of oral mucosa before dental injection is an effective alternative to lidocaine 5% gel.

**Trial registration:**

The European Union Drug Regulating Authorities Clinical Trials Database EudraCT201300530531. Date of registration: February 10th, 2014.

## Background

Despite the continuous development of new dental injection techniques, the injection of local anaesthesia still causes discomfort and pain for many patients and has been described as a major reason for dental anxiety [1–3]. Topical anaesthesia should therefore be used to eliminate or minimize the pain caused by the needle.

Topical anaesthetic gels are common in dentistry [4], but studies have shown varying results on their effects. Topical anaesthesia does not guarantee completely pain-free injections, and the efficacy is dependent on several factors such as time (injection speed) and gauge size of the needle [5]. Some authors have stated that lidocaine 5% gel only relieves pain caused by the needle insertion, but not the pain caused by the actual injection, unless a 10-min period has passed since administration of topical anaesthesia [6]. There are even studies questioning whether topical anaesthesia has any effect on pain during either needle insertion or injection [7].

The methods used today for topical anaesthesia before dental injections mainly include different types of gel (e.g. lidocaine, prilocaine or benzocaine). Unfortunately, these gels have a tendency to spread in the mouth due to lack of bioadhesion, which may result in a reduced anaesthetic effect [8], unpleasant taste, and/or discomfort for the patient [9]. Furthermore, there is a risk of allergic reactions to several components in the different gels available for topical anaesthesia [10]. These factors are important reasons for developing and evaluating other substances and techniques for topical anaesthesia.

Ice was introduced during the nineteenth century as a safe method of local anaesthesia [11], and the use of cooling for reduction of injection pain in the skin is well documented. However, published studies evaluating ice as topical anaesthesia in dentistry are very rare [12]. One study from 1989 described how ice on the palate before and during local infiltration anaesthesia could relieve discomfort, but did not compare ice with other agents for topical anaesthesia [13]. Another study found that cooling increased the efficacy of conventional topical anaesthesia, but did not evaluate ice as the only topical anaesthetic agent [14]. Thus, ice as topical anaesthesia of the oral mucosa is not an established method in dentistry today [12].

Several explanations for the anaesthetic effect of cooling have been proposed [15]. Topical cold application would for instance stimulate myelinated A fibers, thereby activating inhibitory pain pathways [16], perhaps as part of the gate control system at the spinal cord level [15]. Furthermore, cooling causes cold-induced neuropraxia by decreasing the activation threshold of tissue nociceptors and the conduction velocity of nerve signals conveying pain [17, 18].

The aim of this study was to describe a new simple method for topical anaesthesia of oral mucosa and to compare the anaesthetic effect and subjective experience of two topical anaesthetic agents, lidocaine 5% gel and ice, applied on the oral mucosa prior to needle stick and injection of local anaesthesia.

## Methods

The study was approved by the Regional Ethical Review Board in Linköping (ref: 2013/315–31 – December 11th, 2013) and the Swedish Medical Products Agency (EudraCT no: 2013–005305-31 - date of registration of TRN: February 10th, 2014) before the start of the clinical investigations, and the process was monitored by Forum Östergötland, Linköping, Sweden. Our study adheres to the CONSORT guidelines (http://www.consort-statement.org/) for reporting clinical trials.

The clinical part of the study (data collection) was performed by two general dentists at a public dental clinic from August 2014 to August 2017.

### Inclusion criteria

Healthy patients (ASA 1; see [19]), who were aged under 20 years, did not express any dental fear, and were planned for orthodontic treatment including extractions of two contralateral maxillary premolars without pathology, were invited to participate in the study. Participants as well as their legal guardians (for those aged under 18) had to be positive to participate in the study.

### Exclusion criteria

Patients were excluded if they had any medical conditions considered to affect patient safety or the quality of the study, or if they had any known hypersensitivity to local anaesthetics of amide type or any of the other contents in the substance used. If the study protocol was not followed in a way that made it impossible to compare the anaesthetic effect and subjective experience of the two topical anaesthetic agents according to the aim of the study, the patient was excluded from the study. Patients without written consent signed by themselves and by both parents/guardians were excluded. If a patient or parent/guardian denied further participation the patient was immediately excluded from the study without any further questions.

### Information and consent

The orthodontist referred the patients, who were positive to participate, to the principal investigator (PI). All patients, who were suitable according to the inclusion and exclusion criteria, received a phone call from the PI, who informed them further about the study and asked if they were willing to participate in the study. Patients and guardians were informed that they had the right to discontinue their participation in the study at any time and for any reason without having to motivate their decision, and that not participating, or leaving the study, would not influence the remaining or planned treatment. They also received verbal and written information about the study and were encouraged to ask questions about it. If they decided to participate in the trial, they had to bring a consent form to the first treatment session, signed by both the patient and, when applicable, by the legal guardians.

### First appointment

At the first visit, the PI verified that the patient and the legal guardians (when applicable) had received and understood the information given and that they had given their consent to participate in the study. A set of envelopes numbered consecutively by a statistician according to inclusion in the study was used to conduct the randomisation in blocks of four. Before starting the procedure, an envelope was opened to see which side of the maxilla (right or left) and which topical anaesthesia (lidocaine 5% gel or ice) to use at the first and second appointment, respectively.

**Fig. 1:**
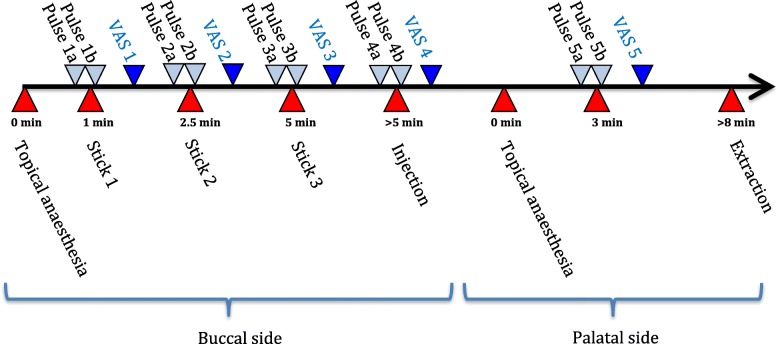
Flow chart describing the time schedule at each visit. Visual analogue scale (VAS) was used for evaluation of pain and discomfort according to the questionnaire presented in Table 1

The procedure for each treatment session is presented in Fig. 1. A pulse oximeter (Contec PM60A, Contec Medical Systems, Qinhuangdao, China) was applied to the patient’s left index finger.

### Technique for application of topical anaesthetics

**Fig. 2:**
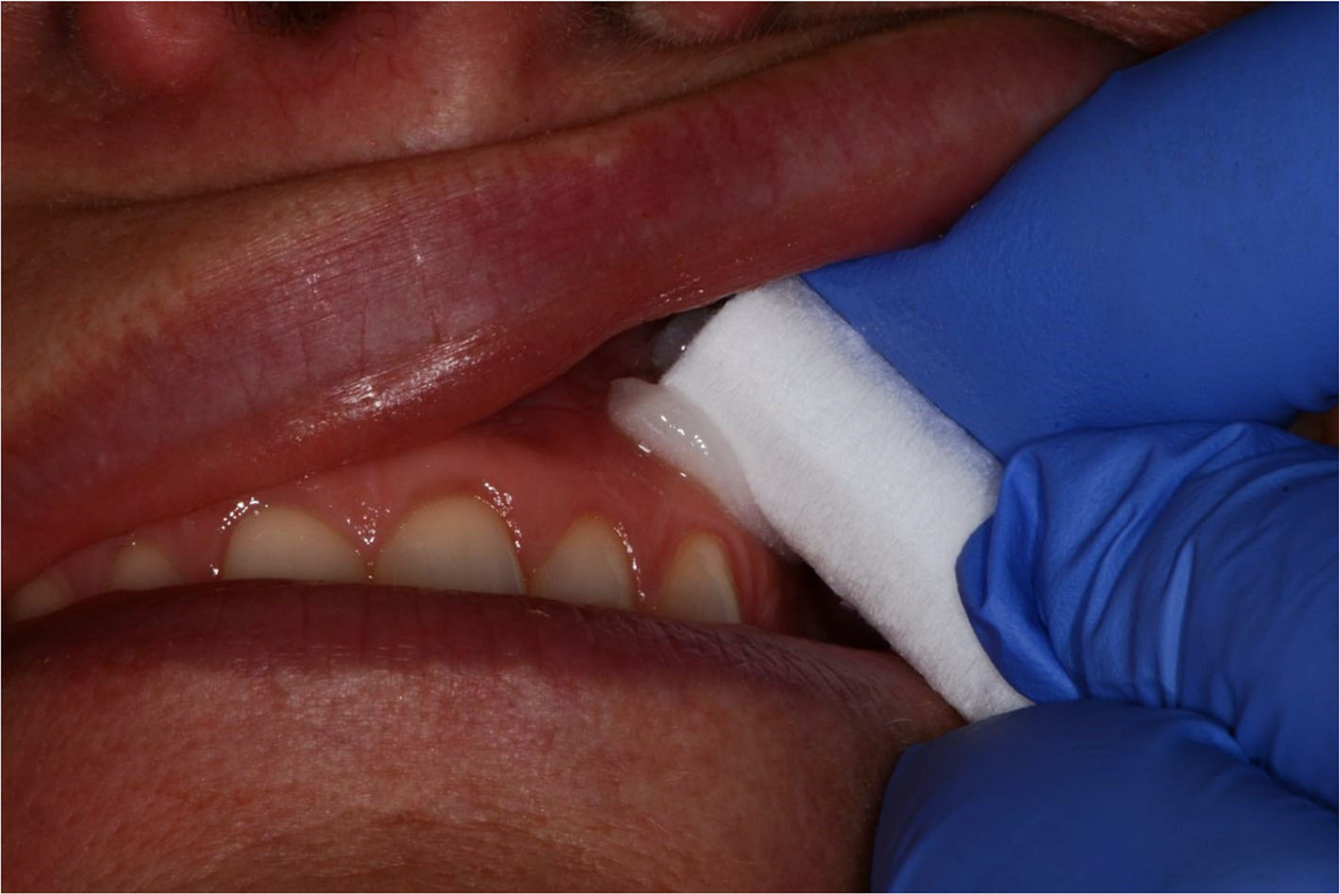
Lidocaine 5% gel applied on the buccal mucosa

**Fig. 3:**
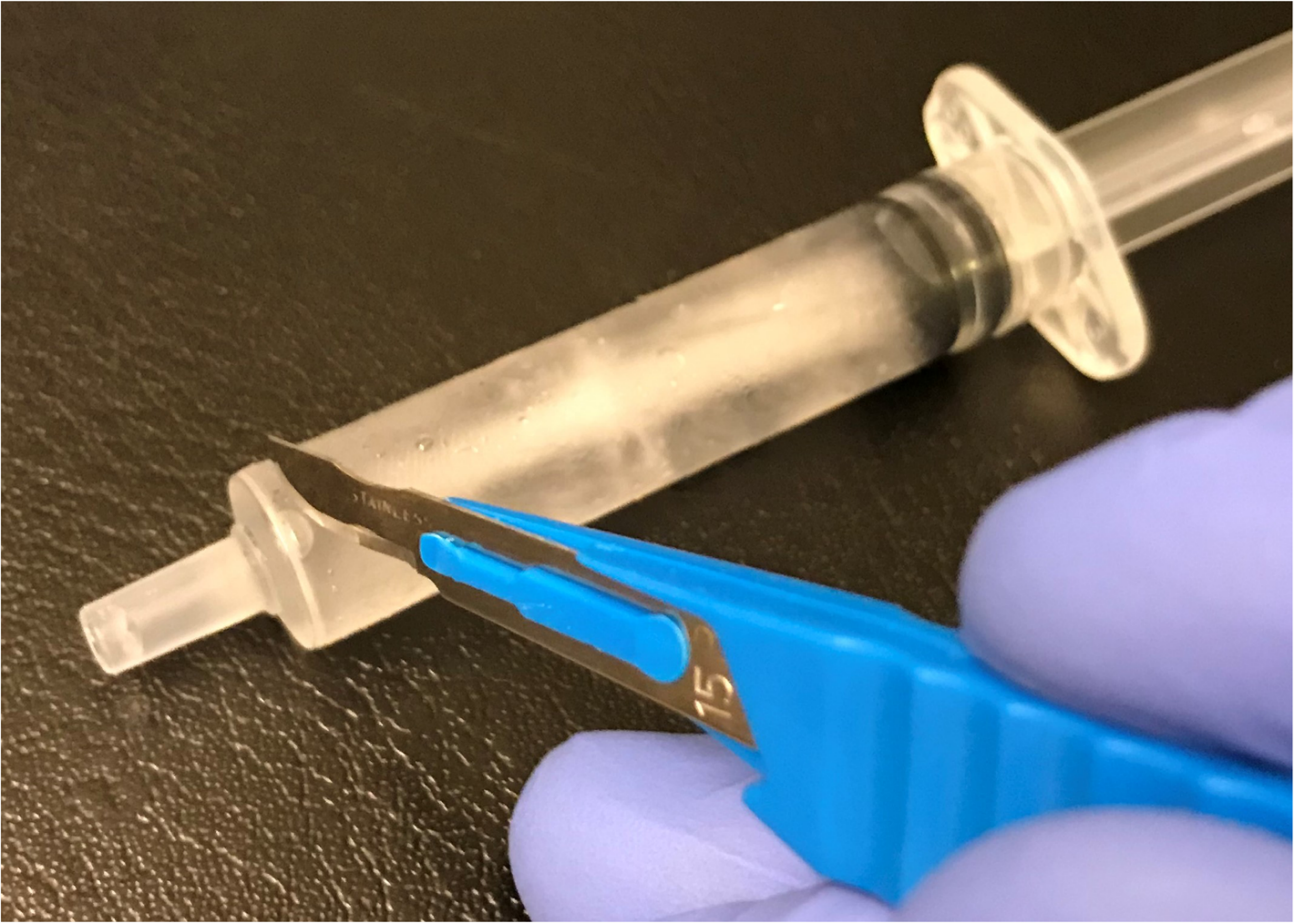
The 2.5 ml syringe was filled with ordinary tap water which was frozen at the clinic. A scalpel was used to cut off the tip of the syringe

**Fig. 4:**
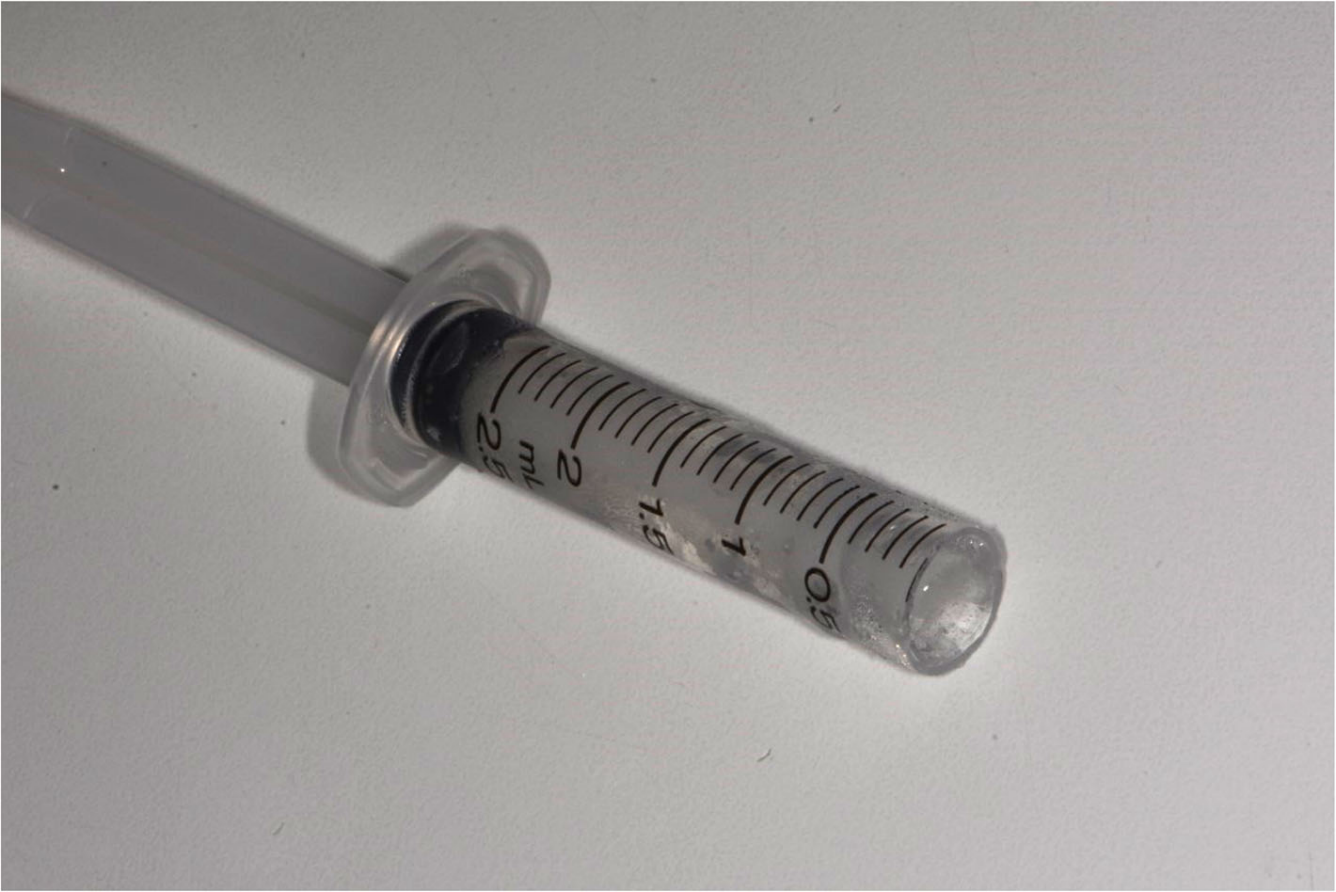
Ice-filled syringe after cutting off the tip

**Fig. 5:**
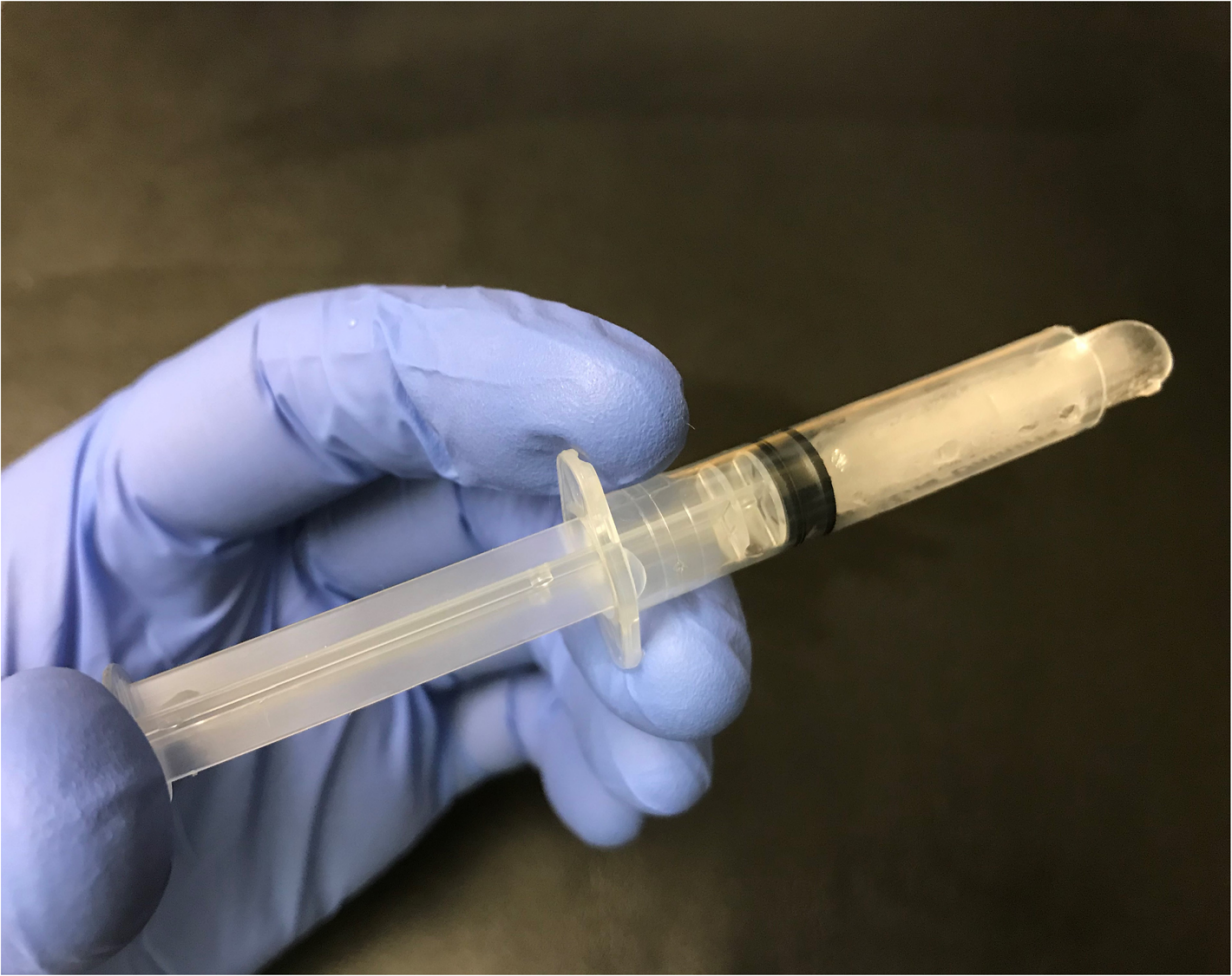
The ice-filled applicator in the hand of the operator

**Fig. 6:**
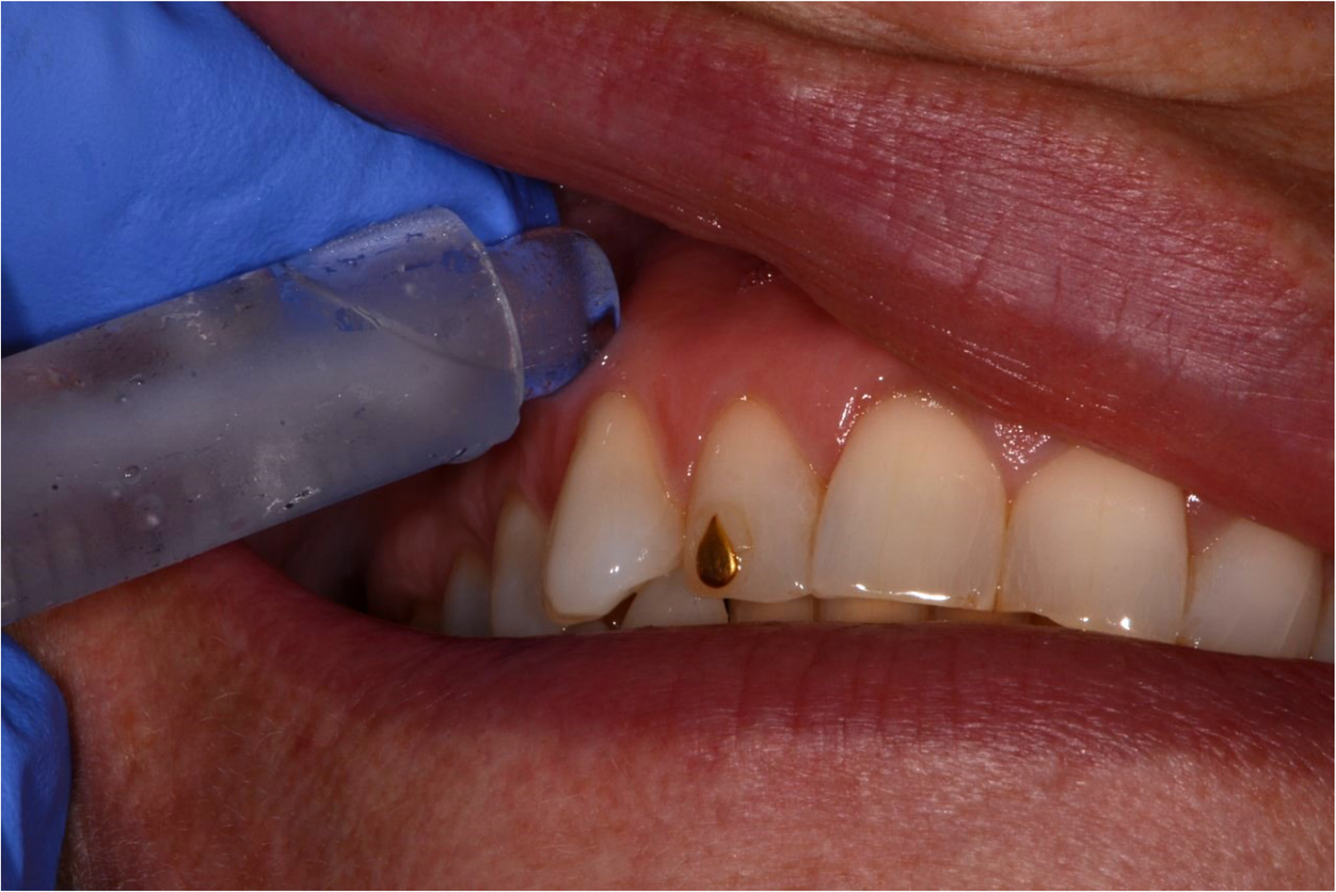
Ice applied on the buccal mucosa

The oral mucosa was dried with a cotton roll before application of the topical anaesthesia. Application of 0.2 mL lidocaine 5% gel (Apoteket, Stockholm, Sweden) was performed with a cotton roll on the oral mucosa (Fig. 2). Ice was prepared by filling a 2.5 ml plastic syringe (BD Plastipak, 2 ml, Ref 300,185, Becton Dickinson, San Agustín del Guadalix, Madrid, Spain) with tap water and freezing it in a freezer at the clinic. The ice temperature was between − 4 °C and 0 °C to avoid any risk of frostbite. Before administration, the tip of the plastic syringe was cut off with a scalpel (Figs. 3 and 4), allowing the ice to be pressed out on the mucosa while it melted (Figs. 5 and 6). The patient answered the questionnaire (Table 1) after each sequence of the study (Fig. 1).

**Table 1:** Questions answered by the patient, following the flowchart presented in Fig. 1. For evaluation of the palatal site, only questions 4–9 were used

1. How painful was the needle stick at 1 min?^a^	
2. How painful was the needle stick at 2.5 min?^a^	
3. How painful was the needle stick at 5 min?^a^	
4. How painful was the injection?^a^	
5. How did the topical anaesthesia feel?^b^	
6. What’s your opinion of the taste of the topical anaesthesia?^c^	
7. Did you feel any discomfort or irritation in the area treated with topical anaesthesia?^d^	
8. If you answered yes to Question 7, please describe what you felt.	
9. Please write something more about what you thought of the pre-treatment with gel or ice.	

^a^Answered on a 100 mm visual analogue scale (VAS) with the extremes ‘no pain’ and ‘worst possible pain’

^b^Answered on a 100 mm VAS with the extremes ‘no discomfort’ and ‘worst possible discomfort’

^c^Answered with the alternatives ‘very good’, ‘good’, ‘no taste’, ‘bad’, ‘very bad’

^d^Answered with the alternatives ‘yes’ or ‘no’

### Buccal evaluation

The effect of the topical anaesthesia on the buccal site was evaluated with a 1 mm deep needle stick (with a 30-gauge ½ inch short needle) apically of the tooth, without bone contact, for 1 s at 1, 2.5, and 5 min, counted from when the topical anaesthesia was first applied to the mucosa. Injection of 1.5 ml Xylocain Dental Adrenalin 20 mg/ml lidocaine + 12.5 micrograms/ml adrenaline (AstraZeneca, Södertälje, Sweden) was performed with the same 30-gauge ½ inch short hypodermic needle applied to The Wand (Milestone Scientific, Livingston, NJ, USA), 5 min after application of topical anaesthesia. The injection was performed with the lowest possible injection speed for The Wand. Pain was graded by the patient on a 100 mm visual analogue scale (VAS) after each stick and after each injection (Table 1). Discomfort was also graded on a VAS scale after injection. The patients reported if there was any irritation in the mucosa, and graded the taste of the topical anaesthesia on a five-point Likert scale (Table 1).

### Palatal evaluation

Topical anaesthesia was not applied to the palatal mucosa until after the injection and evaluation of the buccal side was completed, and was only evaluated after the injection, which was performed 3 min after application of the topical anaesthesia (Fig. 1). The patient answered the same questions for the palatal side as for the buccal side, with the exception of the three needle sticks, since they were not performed on the palatal side (Table 1, Fig. 1).

### Heart rate

Heart rate (HR) in beats per minute (bpm) was registered by the treating clinician at baseline and before and after each needle stick on the buccal side, and after injection on the buccal and palatal side (Fig. 1). The relative HR change (HR immediately after each intervention divided by HR immediately before each intervention) was used for evaluation of HR change during each intervention (insertion or injection).

After these steps, the premolar was extracted, but the extraction procedure was not evaluated in the study. One week after each treatment, the PI called the patient to ask if there had been any unexpected side effects (Table 2).

**Table 2:** Actions performed before, during, and after each visit

Visit no:	1^a^		2		3	
Phone call no:		1		2		3
Verbal information	X	X				
Informed consent			X			
Patient history	X					
Inclusion and exclusion criteria	X		X			
Randomization			X			
Treatment			X		X	
Registration of possible adverse events			X	X	X	X

^a^Performed at the orthodontic clinic

### Second appointment

The second appointment was not performed until all symptoms and possible side effects from the first treatment had definitely subsided. A minimum time of 2 weeks between the extractions was applied, to ensure that no confounding pain from the first extraction persisted at the second visit. At the second appointment, the mucosa at the contralateral maxillary premolar was treated with the topical anaesthesia (lidocaine 5% gel or ice) that had not been used at the first appointment. The same protocol was followed for the second appointment. One week after the second treatment, the PI made another phone call to ask about any unexpected side effects of the treatment. This phone call completed the patient’s participation in the study (Table 2).

### Statistical methods

The primary outcome was whether ice and lidocaine had similar effects on subjective pain according to the VAS, with effects being considered similar if the mean ± standard deviation (SD) difference, δ, between the methods was 4 ± 6 mm or less. A statistical power calculation based on previous results concluded that 40 patients would be needed to achieve 80% power to detect the primary outcome at the 0.05 level of significance when using the paired t-test to compare mean values. Paired t-test was used for evaluation of repeated measures, a chi^2^ test was used for comparison of proportions, and a Pearson correlation test was used for analysis of correlation between variables. Unless stated otherwise, data are expressed as mean ± SD.

## Results

Data were collected from 5 August 2014 to 30 August 2016. Of the 42 patients who agreed to participate, one was excluded because the same topical anaesthesia was accidentally used at both visits, and another was excluded because written consent could not be obtained from one of their parents. The remaining 40 (19 M, 21F) participants had an mean age of 15.6 ± 2.2 years (range: 10.7–19.5) when they entered the study. None of the participating patients or guardians denied further participation after entering the study. The HR change measurements from 13 (6 M, 7F) patients could not be evaluated since they only included pulse at the beginning of each visit and measurements *after* each intervention (insertion or injection), but not immediately *before* each intervention. HR change (from before to after each insertion and injection, respectively) could therefore only be evaluated in 27 (13 M, 14F) of the patients.

The first treatment was performed with ice as topical anaesthesia in half of the patients (*n* = 20) and lidocaine 5% gel in the other half (*n* = 20). None of the participants were smokers, and none expressed any dental fear.

### VAS ratings

The VAS ratings at the different interventions following application of ice and lidocaine 5% gel are presented in Table 3. When ice was used, buccal VAS pain at 2.5 min was rated higher (*p* = 0.016), buccal injection pain was rated lower (*p* = 0.044), and VAS discomfort was rated higher (*p* = 0.001), in comparison to when lidocaine was used. Palatal VAS data did not differ between the two methods (Table 3).

**Table 3:** VAS ratings after the different interventions (needle insertion and injection) following application of ice and lidocaine 5% gel (*n* = 40)

Intervention/variable measured (application time)	Ice mean ± SD (mm)	Lidocaine 5% gel mean ± SD (mm)	Paired *t*-test (*p*-value)
VAS pain buccal needle insertion (1 min)	9.7 ± 9.2	8.5 ± 9.6	0.469
VAS pain buccal needle insertion (2.5 min)	11.8 ± 9.4	7.3 ± 10.4	0.016
VAS pain buccal needle insertion (5 min)	11.8 ± 13.7	8.2 ± 7.5	0.079
VAS pain buccal injection	12.4 ± 10.6	16.4 ± 14.5	0.044
VAS discomfort buccal injection	9.6 ± 10.7	3.7 ± 3.7	0.001
VAS pain palatal injection	19.1 ± 11.0	21.4 ± 14.17	0.252
VAS discomfort palatal injection	6.5 ± 8.5	5.9 ± 9.1	0.633

When comparing VAS pain ratings following needle insertion at different times after application of the topical anaesthesia, there were no differences between ratings at 1 min (ice: 9.7 ± 9.2; gel: 8.5 ± 9.6) and at 2.5 min (ice: 11.8 ± 9.4; gel: 7.3 ± 10.4) for either ice (*p* = 0.168) or lidocaine 5% gel (*p* = 0.382). Similarly, there were no differences between ratings at 1 min and at 5 min (ice: 11.8 ± 13.7; gel: 8.2 ± 7.5) for either ice (*p* = 0.242) or gel (*p* = 0.832).

VAS pain ratings were higher for the palatal injection (ice: 19.1 ± 11.0; gel: 21.4) than for the buccal injection (ice: 12.4 ± 10.6; gel: 16.4 ± 14.5), and according to the paired t-test the difference was significant both when ice was used (*p* = 0.000) and when lidocaine 5% gel was used (*p* = 0.008). When ice was used, the VAS discomfort rating was higher (*p* = 0.011) following buccal injection (9.5 ± 10.7) than following injection on the palatal site (6.5 ± 8.5). When lidocaine 5% gel was used, there was no difference in discomfort between buccal and palatal injection.

There were no correlations between age and either VAS pain or VAS discomfort.

For treatment on the buccal site, a significantly higher proportion of patients (*p* = 0.025; chi^2^ test) reported a bad or very bad taste when lidocaine 5% gel was used (*n* = 7; 17.5%) than when ice was used (*n* = 1; 2.5%). The situation was similar on the palatal site, with a significantly higher (*p* = 0.018; chi^2^ test) proportion of patients reporting a bad or very bad taste when lidocaine was used (*n* = 14; 35%) than when ice was used (*n* = 5; 12%).

There were no gender differences regarding the prevalence of a bad or very bad taste for either the ice or the gel on the buccal or palatal site. There were also no significant gender differences in VAS ratings, except that girls’ VAS pain ratings following injection were higher than boys’ for both the buccal (girls: 15.7 ± 13.1; boys: 12.9 ± 12.4; *p* = 0.004) and palatal (girls: 23.1 ± 13.1; boys: 17.2 ± 11.4; *p* = 0.032) sites.

None of the VAS ratings regarding pain, discomfort, or taste differed significantly between the first and second visit.

### Heart rate

The initial HR at the start of the first visit was 83 ± 15 bpm and did not differ significantly (*p* = 0.14) between girls (86 ± 15 bpm) and boys (79 ± 13 bpm); it also did not differ significantly (*p* = 0.417) from the initial HR at the second visit (80 ± 15 bpm). There was a negative (Pearson) correlation (*p* = 0.001) between baseline pulse at first visit and age (*r* = − 0.50).

The relative HR change at intervention did not differ between lidocaine 5% gel and ice at any of the interventions (insertion of needle or injection) (Table 4).

**Table 4.** Relative heart rate change (heart rate immediately after each intervention divided by heart rate immediately before each intervention) during each intervention (insertion or injection) following application of ice or lidocaine 5% gel (*n* = 27)

Intervention/variable measured (application time)	Ice mean ± SD	Lidocaine 5% gel mean ± SD	Paired *t*-test (*p*-value)
HR change buccal needle insertion (1 min)	0.99 ± 0.04	0.98 ± 0.04	0.665
HR change buccal needle insertion (2.5 min)	0.99 ± 0.06	1.00 ± 0.04	0.506
HR change buccal needle insertion (5 min)	1.00 ± 0.07	1.00 ± 0.04	0.706
HR change buccal injection	1.00 ± 0.07	1.02 ± 0.08	0.210

*HR* heart rate

There was no significant difference (*p* = 0.636) between the relative HR change following injection on the buccal (1.00 ± 0.07) and palatal side (0.99 ± 0.07) following application of ice, while the application of lidocaine 5% gel resulted in a HR change significantly different (*p* = 0.010; paired t-test) between palatal and buccal site, with a relative reduction after palatal injection (0.99 ± 0.06) as compared to a relative increase after buccal injection (1.02 ± 0.08).

### Comments from patients

Of the 30 patients who wrote comments in their final questionnaire, seven found the two methods similar, eight preferred the lidocaine 5% gel, and 15 preferred the ice. The preferred method was not correlated with the method used at the last appointment. One of the patients who found no difference between the two methods did comment that the ice resulted in more water in the mouth. Five patients, all of whom preferred ice to gel, commented on a bad taste. Four patients found the ice more comfortable than the gel. One said that the treatment time felt shorter when ice was used. Better anaesthetic effect was mentioned by four patients who preferred the lidocaine 5% gel and three who preferred the ice. No unintended side effects were found.

## Discussion

Pain has been defined by the International Association for the Study of Pain as “an unpleasant sensory and emotional experience associated with actual or potential tissue damage, or described in terms of such damage” [20]. The purpose of using topical anaesthesia before dental injection is to eliminate or reduce the pain as effectively and non-invasively as possible. Although previous authors have questioned the effect of topical anaesthesia before injection in the oral cavity [7], many studies have shown a good effect [5, 6, 21, 22].

A confounding factor in the study of pain and discomfort is that the sensation and feelings are subjective, leading to difficulties in objectively comparing experiences of pain and discomfort from different individuals [23]. We therefore decided to compare pain from one side to the other in the same patient. Lidocaine 5% gel was used as a control substance to compare with the ice, since its effect has been described earlier [21, 22] and it is commonly used as topical anaesthesia in dentistry [4]. To minimize the discomfort and pain, and to standardise the injection speed, we used a 30-gauge ½ inch needle along with a computerised standardised anaesthetic injection device, The Wand, as described in a previous study [24].

We decided to use pulse change and VAS ratings as indicators of pain and discomfort, as these have been found to be reliable indicators of a patient’s response to pain [23, 25] and have also been used for evaluation of discomfort [24, 26]. The study was designed as a randomized split-mouth crossover study, which reduced the risk of bias since each patient received both treatments. This gives more reliable results, since there is a variability between different patients’ response to pain [27]. To minimize the risk for bias related to the evaluation of the buccal site, topical anaesthesia on the palatal site was not applied until after the completion of injection and evaluation of the buccal side.

In 2009 Aminabadi and Farahani [14] stated that only one previous study [13] had investigated the effects of local mucosal cooling prior to infiltration of local anaesthesia in dentistry. Even if later published studies have shown results indicating that pre-cooling is effective, the pre-cooling can be administered in many different ways and in combination with various other techniques. An article from 2015 [16] stated that even if the use of cryoanesthesia to reduce injection pain had been reported to be promising, only sparse literature reports exist regarding the clinical efficacy of these agents. Our study is an effort to increase the available publications in this field of knowledge.

Many of the published articles dealing with pre-cooling of oral mucosa prior to dental injection have used ice in combination with pharmaceutical topical anesthetics [28–30], why the genuine effect of the cooling has not been evaluated in those studies, as it was done in our study.

The study design had two main limitations. Firstly, it was unblinded, which always increases the risk of bias. Unfortunately neither patients nor clinicians could have been blinded, since the temperature difference between lidocaine 5% gel and ice, and the different methods of application, made it obvious which method was used. Secondly, although a control group treated with placebo could have made the results even stronger, we felt that ethical considerations ruled this out as an option, since we did not wish to cause any painful experience that could increase the risk of dental anxiety.

The VAS is a frequently used reproducible method for the measurement of painful procedures [23, 31, 32]. The only difference between the genders was that the girls scored the VAS pain at injection higher than boys, which is in accordance with several other reports [2, 24, 33]. Generally speaking, the VAS pain intensity ratings in our study were low, for both ice and lidocaine gel and at all time points, when compared to medical pain ratings [34]. Several previous studies – using ice as a pre-cooling agent before dental injection - have only evaluated a shorter time for application of the cooling agent; usually 1 min [16, 28–30]. In our study the ice was kept in place during 5 min before injection was performed, and evaluation of insertion pain was measured three times during that time. Our results showed that there was no additional reduction of insertion pain after a 1-min application time for either ice or gel, which makes it reasonable to suggest that for both these methods an application time of 1 min is sufficient to reach superficial topical anaesthetic effect before injection using a 30 gauge needle. An earlier study concluded that lidocaine 5% gel might provide a better superficial anaesthesia of the oral mucosa if the application time was extended to 15 min [24], although the recommended application time on the summary of product characteristics is 2–3 min [35]. Our findings suggest that this application time can be reduced to 1 min, but since this is only applicable to the superficial insertion pain, it is necessary to perform the injection gradually and very slowly to avoid pain from tension in the tissue. It is also important to slowly increase the depth of the needle into already anaesthetized tissue before further injection of local anaesthesia can be performed by stages, in order to make the injection as pain-free as possible.

The finding that a palatal injection was more painful than a buccal injection is well known to dental clinicians, and is probably due to the absence of free mucosa on the palatal side. The fact that the mucosa of the hard palate is highly keratinized and very dense and tight [36] makes the injection pain from pressure in the tissue more prominent. Several studies have reported failure to satisfactorily anaesthetize the palatal site [37–40].

Our finding that a significantly higher proportion of the participants reported a bad or very bad taste following treatment with lidocaine 5% gel on the buccal site, in comparison to when ice was used on the same site, confirms that the patient’s experience of topical anaesthesia can be improved if a different substance is used or if the method for application is improved. Previous studies have also emphasized the bad taste experience associated with anaesthetic gels [41].

The simple method for topical anaesthesia of oral mucosa (using a plastic syringe filled with frozen water for direct application of ice on the oral mucosa) described in our manuscript has not been published previously, and an evaluation of the comparison between this method and Lidocain 5% gel for topical anaesthesia of the oral mucosa has therefore not been presented heretofore. The methods used by several other authors have included ice incapsulated in plastics [14, 29] or in empty cartridges of glass filled with ice [30] or ice-filled cotton buds [28]. All these mentioned methods have the disadvantage of not having direct contact between the ice and the mucosa. Under such circumstances the plastics or glass will isolate the ice from the surrounding. The superficial part of the ice will melt first and melted water will surround the ice (when covered by plastics or glass), diminishing the temperature effect from the ice on the oral mucosa after a short time of application. When an ice-filled cotton roll or cotton bud is used, the ice on the surface will melt first and after a short time there will be no ice in direct contact with the oral mucosa. The temperature on the oral mucosa will therefore be higher for all those other methods than when ice is in direct contact with the oral mucosa. The method described in our study makes it possible to have direct contact between the ice and the oral mucosa during the entire time of application (Fig. 6).

In a study by Lathval et al. [16] a custom made ice cone was used for topical anaesthesia of the injection site, but the size and shape of the ice cone was not described, neither how the ice cone was kept in place [16]. Jayasuriya et al. [28] stated that a limitation of the method when ice is placed directly on the oral mucosa, is that ice will slip from the operators hand due to wetness and will cool the operators fingers [16]. These problems are, anyhow, not present when our described method is used, since the operator’s fingers are only in contact with the plastic syringe and will not touch the ice itself (Figs. 5 and 6).

No previous study has presented the temperature of the ice used for cooling of the oral mucosa. The temperature of the cooling agent is important, since too low temperature or too long application time may cause frostbite to the oral mucosa – especially if other cooling agents than ice are used [16].

The method described for application of ice on oral mucosa is readily available, and according to the dentists in our study is easy to perform. It could therefore be a good alternative to the commercially available lidocaine 5% gel, especially when other types of topical anaesthesia are not available, or if certain pharmacological components should be avoided due to the risk of allergic reactions or intoxication [10, 42].

Regarding heart rate, the negative Pearson correlation between baseline pulse and age confirmed the findings of previous studies that HR decreases with age [43].We chose to evaluate the relative HR change rather than the actual HR change, to avoid bias from diverging HR at baseline. Unfortunately, the HR from 13 patients was not measured immediately before each intervention, and so their values could not be included in the evaluation of relative HR change. The results showed no significant difference in relative HR change between ice and lidocaine 5% gel, meaning that the two methods have a similar effect on HR change. The significantly larger reduction in relative HR when using lidocaine 5% gel after palatal injection, compared to buccal injection, indicates that the gel had a better pain-reducing effect on the palatal mucosa than on the buccal mucosa. When using ice, there was no significant difference in HR change following injection between the buccal and palatal sites, indicating that topical anaesthesia with ice had similar pain-relieving effects on both the buccal and palatal mucosa. A limitation of the HR analysis was that data were only available from 27 of the 40 patients. However, we believe that this is an acceptable number of patients from which to draw conclusions, since previous similar studies have also included around 30 patients [24, 25, 37, 44, 45]. Another limitation is that HR is a very unspecific metric, and that it can e.g. be influenced by memories or emotional reactions, not least related to smell [46].

The individual comments by the patients showed an obvious variety of preferences. While the effectiveness of the topical anaesthesia was important to several patients, comfort was more important to others. This implies that a continuous communication with the patient is important in choosing the best method of topical anaesthesia for each individual.

## Conclusions

The described method for the use of ice as topical anaesthesia on oral mucosa before injection has an effect similar to that of lidocaine 5% gel on pain relief at insertion of needle, and the topical anaesthetic effect is present as early as 1 min after application. The taste of lidocaine 5% gel is worse than that of ice. The method described here for administration of ice as topical anaesthesia before injection in oral mucosa is a good, cheap, easy available alternative to the commercially available lidocaine 5% gel.

## Data Availability

The datasets used and/or analysed during the current study are available from the corresponding author on reasonable request.

## Abbreviations

BPM: beats per minute
HR: Heart rate
PI: Principal investigator (PI)
VAS: Visual Analogue Scale
